# Skin Biopsy in the Context of Dermatological Diagnosis: A Retrospective Cohort Study

**DOI:** 10.1155/2014/734906

**Published:** 2014-01-30

**Authors:** Chrysovalantis Korfitis, Stamatis Gregoriou, Christina Antoniou, Andreas D. Katsambas, Dimitris Rigopoulos

**Affiliations:** ^1^Department of Dermatology, Veterans Administration Hospital, 10-12 Monis Petraki Street, 11521 Athens, Greece; ^2^Department of Dermatology, Attikon Hospital, 1 Rimini Street, Haidari, 12462 Athens, Greece; ^3^Department of Dermatology, Andreas Sygros Hospital, 5 I. Dragoumi Street, 16121 Athens, Greece

## Abstract

*Background*. Skin biopsy is an established method for allying the dermatologist in overcoming the diagnostic dilemmas which occur during consultations. However neither do all skin biopsies produce a conclusive diagnosis nor the dermatologists routinely perform this procedure to every patient they consult. The aim of this study was to investigate the favourable clinical diagnoses set by dermatologists when performing skin biopsy, the diagnoses reached by the dermatopathologists after microscopic examination, and the relationship between them and finally to comment on the instances that skin biopsy fails to fulfill the diagnostic task. *Methods*. Six thousand eight hundred and sixteen biopsy specimens were reviewed and descriptive statistics were performed. *Results*. The mean age of the patients was 54.58 ± 0.26 years, the most common site of biopsy was the head and neck (38.3%), the most frequently proposed clinical diagnoses included malignancies (19.28%), and the most prevalent pathological diagnosis was epitheliomas (21.9%). After microscopic examination, a specific histological diagnosis was proposed in 83.29% of the cases and a consensus between clinical and histological diagnoses was observed in 68% of them. *Conclusions*. Although there are cases that skin biopsy exhibits diagnostic inefficiency, it remains a valuable aid for the dermatology clinical practice.

## 1. Introduction

The management of skin diseases requires a pertinent diagnosis, which in many occasions constitutes an intricate process. Skin biopsy is an established diagnostic procedure which connects clinical diagnostic methodology with the invisible to the unaided eye microscopic field of skin pathology. Taking under consideration the potentials and limitations of optical microscopy and the indications of performing an invasive technique, dermatologists often rely on skin biopsy for enhancing their diagnostic abilities. The aim of this study was to investigate the favourable clinical diagnoses set by dermatologists when performing skin biopsy, the diagnoses reached by the dermatopathologists after microscopic examination, as well as the relationship between them, and finally to comment on the instances that skin biopsy fails to fulfill the diagnostic task.

## 2. Methods

Six thousand eight hundred and sixteen (6816) biopsies were reviewed which were included in 5941 histopathology report forms and were processed in the “Andreas Sygros” hospital during the years 2004–2006. Furthermore, a topographic anatomy coding system was developed along with an ad hoc coding system for skin diseases in order to meet the requirements of the study (data not shown). Each of the 5941 patients underwent at least one and at most seven skin biopsies at any session. The frequencies of the various sites of biopsy, the percentages of all clinical diagnoses proposed by the dermatologists, and the percentages of the histological diagnoses set by the dermatopathologists were calculated and statistical significance was evaluated.

Data were analyzed using PASW Statistics version 18 (SPSS Inc, Chicago IL). Descriptive statistics were applied including frequencies and percentages, as well as the chi-square test, both for one-way and contingency tables. The level of significance was set at less than 0.05.

## 3. Results

### 3.1. Gender and Age

Out of 5941 patients that underwent skin biopsy, 48.2% (*n* = 2862) were males and 51.8% (*n* = 3075) females, with *n* = 4 missing data. The mean age was 54.58 ± 0.26 and the median 57 years. The mean age for males was 56.54 ± 0.37 (median 60 years) and for females 52.79 ± 0.36 (median 54 years).

### 3.2. Site of Biopsy

The site of each biopsy was studied regarding both anatomic regions and specific locations. Regarding anatomic regions, the respective frequencies were found to be the head and neck 38.3% (*n* = 2515), the anterior and lateral tegument 14.3% (*n* = 941), the posterior tegument 12.3% (*n* = 810), the pelvis 6.9% (*n* = 454), the upper extremities 11.1% (*n* = 729), and the lower extremities 17.1% (*n* = 1124). Out of 6573 valid biopsy sites (*n* = 243 missing) the most common specific locations were the back 8.8% (*n* = 579), the scalp 5.9% (*n* = 387), the nose 3.3% (*n* = 218), and the abdomen 3.3% (*n* = 217). After performing the chi-square test, the differences in the frequencies were found statistically significant (*χ* ^2^ = 2434.521, *P* < 0.001).

### 3.3. Clinical Diagnoses

**Figure 1:**
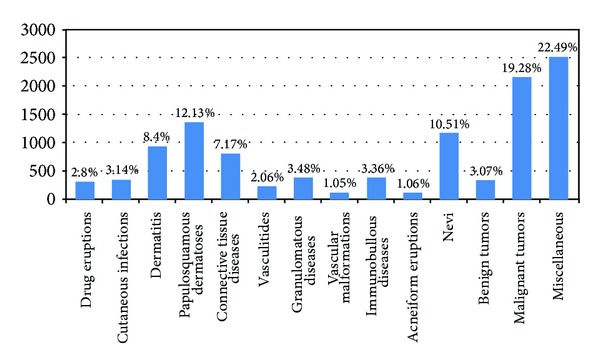
Bar chart of a classification of all the proposed clinical diagnoses.

In order to study the clinical diagnoses that were proposed by the dermatologists, 6733 out of 6816 biopsies (98.78%) were evaluated. There were 11194 valid specific diagnoses, divided in 367 different terms of skin diseases, after excluding clinical descriptions and intangible expressions (12579 in total, 13 of which referring to different tissue other than skin), producing a ratio of 1.66 proposed diagnoses per skin biopsy. No diagnosis at all was given in *n* = 158 cases (2.4%). A classification of 14 categories of all clinical diagnoses is presented in Figure 1. The respective frequencies were “malignant tumors” *n* = 2158 (19.28%), “papulosquamous dermatoses” *n* = 1358 (12.13%), and “nevi” *n* = 1176 (10.51%) including melanocytic nevi, congenital nevi, Spitz nevi, blue nevi, dysplastic nevi, junctional-compound-intradermal nevi, nonmelanocytic nevi, and epidermal nevi, all expressed forms of “dermatitis” *n* = 941 (8.4%) including dermatitis, contact dermatitis, acute or chronic dermatitis, dyshidrotic eczema, nummular eczema, atopic dermatitis, and seborrheic dermatitis; also, “connective tissue diseases” *n* = 803 (7.17%), noninfectious granulomas and “granulomatous diseases” *n* = 389 (3.48%), “immunobullous diseases” *n* = 376 (3.36%), “cutaneous infections” *n* = 351 (3.14%), “benign tumors” *n* = 344 (3.07%), “drug eruptions” *n* = 314 (2.8%), “vasculitides” *n* = 230 (2.06%), acne and “acneiform eruptions” *n* = 119 (1.06%), hemangiomas and “vascular malformations” *n* = 118 (1.05%), and miscellaneous dermatoses *n* = 2517 (22.49%). After applying the chi-square test, the differences in the frequencies were found statistically significant (*χ* ^2^ = 9396.640, *P* < 0.001).

Out of the 367 different clinical expressions, the most common specific diagnoses were “basal cell carcinoma” 9.3% (*n* = 1037), “melanocytic nevus” 7.9% (*n* = 880), “dermatitis” 6.1% (*n* = 685), “plaque psoriasis” 4.6% (*n* = 515), and “squamous cell carcinoma” 3.9% (*n* = 436).

### 3.4. Histological Diagnoses

**Figure 2:**
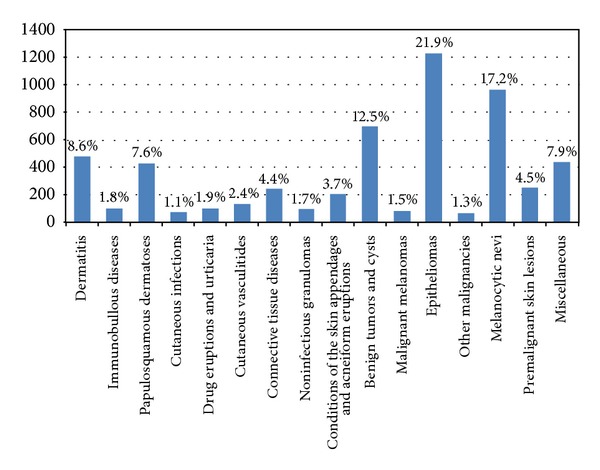
Bar chart of a classification of all the suggested histological diagnoses.

The study of the histological diagnoses that were produced by the dermatopathologists included 6720 skin biopsies (6733 in total, excluding 13 other than skin) and their distinctive pathology that were previously diagnosed clinically by the dermatologists. Five thousand five hundred and ninety-seven (5597) specific histological diagnoses were suggested (83.29%), divided in 259 different terms of skin diseases. A classification of 16 categories of all histological diagnoses is presented in Figure 2. The frequencies and percentages were “epitheliomas” *n* = 1224 (21.9%) comprising basal cell carcinoma, squamous cell carcinoma, basosquamous carcinoma, collision tumors with any epithelioma as a component (e.g., with seborrheic keratosis), keratoacanthoma, fibroepithelioma, and lymphoepithelioma-like carcinoma of the skin, “melanocytic nevi” *n* = 965 (17.2%), “benign tumors and cysts” *n* = 700 (12.5%), “dermatitis” *n* = 484 (8.6%), “papulosquamous dermatoses” *n* = 425 (7.6%), “premalignant skin lesions” *n* = 253 (4.5%), “connective tissue diseases” *n* = 244 (4.4%), “conditions of the skin appendages and acneiform eruptions” *n* = 205 (3.7%), “cutaneous vasculitides” *n* = 135 (2.4%), “drug eruptions and urticaria” *n* = 106 (1.9%), “immunobullous diseases” *n* = 101 (1.8%), “noninfectious granulomas” and granulomatous diseases *n* = 99 (1.7%), “malignant melanomas” *n* = 84 (1.5%), “other malignancies” besides melanomas and epitheliomas *n* = 70 (1.3%), “cutaneous infections” *n* = 61 (1.1%), and miscellaneous dermatoses *n* = 441 (7.9%). Performing the chi-square test, the differences in the frequencies were found statistically significant (*χ* ^2^ = 5150.109, *P* < 0.001). Also, applying the test for contingency tables, the site of biopsy and histological diagnosis were found dependent (*χ* ^2^ = 2917.638, *P* < 0.001) with the most important associations being between epitheliomas followed by conditions of the skin appendages in the head and neck region, cutaneous vasculitides on the lower extremities, melanocytic nevi on the posterior tegument, and dermatitis on the anterolateral tegument.

Among the 259 different expressions used by the dermatopathologists, the most common were “junctional, compound, and intradermal nevi” *n* = 903 (16.1%), “basal cell carcinoma” *n* = 858 (15.3%), and “squamous cell carcinoma” *n* = 304 (5.4%).

### 3.5. Relationship between Clinical and Histological Diagnosis

As mentioned before, 5597 specific histological diagnoses were proposed regarding the underlying pathology of 6720 skin biopsies. In the remaining cases, either a differential diagnosis was offered or no particular suggestion was made. In order to assess the relationship between clinical and histological diagnoses, a classification of ten cases that occurred was employed and for that purpose a separate evaluation was made. The observed frequencies and percentages along with the description of the ten cases are presented in Table 1. A specific histological diagnosis was provided in *n* = 5597 instances (83.3%), no specific histological diagnosis in *n* = 754 (11.2%), whereas two or more were proposed in *n* = 369 (5.5%) of the cases. Useful data orientating the dermatologist in establishing a final clinical diagnosis (cases 1–4 and 7–10 in Table 1) was provided in *n* = 6533 (97.2%) of all biopsies. Histological and clinical diagnoses were found substantially consistent (cases 2, 3, 8, and 10 in Table 1) in *n* = 4571 (68%) of instances. The dermatologists did not provide any specific clinical diagnosis (cases 1 and 6) in *n* = 232 (3.5%) of instances. Moreover, the lack of a specific clinical diagnosis combined with the absence of usable histological data (case 6 only) occurred in *n* = 25 (0.4%) of all cases. With the chi-square test the differences in the frequencies between the cases were found statistically significant (*χ* ^2^ = 10212.560, *P* < 0.001). There was also a dependence of the relationship between clinical and histological diagnosis with the site of biopsy (*χ* ^2^ = 378.979, *P* < 0.001), By interpreting the adjusted residuals, it was found that the correlation lied mostly between case 3 (as described in Table 1) and the biopsy site of the posterior tegument. Also between case 2 and the head and neck region, as well as case 7 and biopsies taken from the pelvis. Also the biopsies from head and neck and the anterolateral and posterior tegument showed a higher consistency between clinical and histological diagnosis.

In Table 1, cases 5 to 7 summarize *n* = 754 skin biopsies with no specific histological diagnosis. Possible reasons that resulted in this difficulty were extrapolated after reviewing the histopathology report forms and classifying the dermatopathologists' comments. Out of a total of 754 biopsies, *n* = 91 (12.1%) specimens were considered as destructed or inappropriate for microscopic examination and *n* = 39 (5.2%) were found of inadequate quantity, where in *n* = 27 (3.6%) the site of biopsy was regarded as not representative or adjacent to the examined lesion, in *n* = 24 (3.2%) the pathological features were altered due to previous treatment, in *n* = 23 (3.1%) optical microscopy with standard staining was considered as inappropriate for a specific diagnosis, and in *n* = 16 (2.1%) the examined lesion was identified as either not fully developed or resolved. The remaining 534 (70.8%) cases were documented as not pathognomonic and without exhibiting distinctive features.

## 4. Discussion

There are many occasions in which a clinician is challenged by a strenuous diagnostic problem. Skin biopsy constitutes a simple and inexpensive procedure performed in the dermatology setting which facilitates clinical decisions regarding diagnosis and treatment. Also, various studies consider histological confirmation as the standard for the correct diagnosis in dermatology as compared to the clinical evaluation, and the results produced in such manner are used in determining the epidemiological characteristics and patterns of skin diseases [1, 2]. Therefore, high diagnostic accuracy is pursued which relies upon the minimization of factors such as inappropriate choice of the lesion, poorly executed technique, unspecified clinical diagnosis and insufficient clinical information, faulty tissue fixation and processing, improper staining for specific diagnoses, or inadequate cooperation between the dermatologist and the dermatopathologist [3–5]. Furthermore, the diagnostic accuracy can be enhanced by using dermoscopy when selecting the site of biopsy [6] and additionally applying immunohistochemical staining and immunofluorescence techniques when appropriate [7, 8].

A few studies have been conducted in order to assess the diagnostic accuracy of skin diseases by physicians by comparing the clinical to the histological diagnosis. One of these studies measured the diagnostic yield of nondermatologists between 34% to 45% and that of dermatologists being 71% and 75% for inflammatory dermatoses or neoplasms and cysts, respectively [9]. Another study found 76.8% of pathological diagnoses to be consistent with the ones given by the dermatologists [10], whereas a third one measured a clinicopathological agreement of up to 92% with this success being attributed by the author to the close cooperation between the dermatologist and the pathologist [2]. In the present study, which was the largest of this kind to our knowledge, a 68% consistency of clinical and histological diagnoses was observed which is lesser than but in accord with the published data. Moreover, further data produced by this study comprise that a specific histological diagnosis was provided in 83.3% of all cases and usable information for the dermatologists was offered in 97.2% of all biopsies.

The data presented herein supports the empirically acquired knowledge of every dermatologist that although skin biopsy is performed for the diagnosis of a wide range of dermatoses, it is used predominantly for the determination of malignancies, mainly melanomas and epitheliomas, and also for inflammatory dermatoses such as dermatitis and psoriasis. Nevertheless, despite the high diagnostic usefulness of skin biopsy (97.2% in this study) with a diagnostic accuracy of 83.3%, there have been 11.2% of all instances lacking histological diagnosis. The possible reasons for this discrepancy have not been quantitatively assessed in the literature. Technically speaking, this could be attributed to several factors such as inadequate and inappropriate specimens, as previously analyzed. However, a number of *n* = 25 (0.4%) of all skin biopsies were lacking both clinical diagnosis and usable histological data. Also there were *n* = 232 (3.5%) without specific clinical diagnosis and *n* = 158 (2.4%) without any clinical description or diagnosis. Although these cases were infrequent, they would probably cause therapeutic problems. Hence, a closer cooperation between the dermatologist and the dermatopathologist is advisable.

## 5. Conclusion

Despite the fact that a plethora of modern techniques have been developed and utilized in the diagnosis of skin disease, dermatologists still rely vastly on biopsy for diagnostic purposes. As discussed in this study, there is a wide range of diseases that allow dermatologists to select skin biopsy in order to confirm their suspected diagnosis, and the histological perspective proves to be both helpful and reliable in the majority of cases. However, there are also limitations in this method and there are cases that the performance of a biopsy does not produce diagnostic results. As a consequence proper diagnosis is delayed and all imminent therapeutic decisions rely heavily upon the dermatologist's comprehension of the situation. Therefore an optimal use of the process is suggested with comprehensive descriptions and relevant diagnoses by the dermatologist along with a closer cooperation with the dermatopathologist performing clinicopathological correlation whenever possible.

## Figures and Tables

**Table 1:** Frequencies and percentages of a classification describing the relationship between clinical and histological diagnoses.

Case	Frequency	Percentage
Case 1: one specific histological diagnosis inconsistent with the unspecified clinical diagnoses	207	3.1
Case 2: one specific histological diagnosis consistent with one specific clinical diagnosis	2642	39.3
Case 3: one specific histological diagnosis consistent with at least one clinical diagnosis regarding the disease category	1668	24.8
Case 4: one specific histological diagnosis inconsistent with the specific clinical diagnoses	1080	16.1
Case 5: no specific histological diagnosis without usable features inconsistent with the specific clinical diagnoses	162	2.4
Case 6: no specific histological diagnosis inconsistent with the unspecified clinical diagnoses	25	0.4
Case 7: no specific histological diagnosis but with usable features, inconsistent with the specific clinical diagnoses	567	8.4
Case 8: two or more specific histological diagnoses constituting subset of the proposed clinical diagnoses	50	0.7
Case 9: two or more specific histological diagnoses different from the proposed clinical diagnoses	108	1.6
Case 10: two or more specific histological diagnoses exhibiting partial overlap with the proposed clinical diagnoses	211	3.1

Total	6720	100.0
